# Supplementary material for: Polymicrobial detection and salivary metabolomics of children with early childhood caries
Source: PeerJ. 2025 May 6;13:e19399. doi: 10.7717/peerj.19399 (PMC12063607; doi:10.7717/peerj.19399)
Supplement: Supplemental Information 3 [file peerj-13-19399-s003.docx]

Method

Sample preparation:
1. Pool QC (PQC) sample preparation: Mix 10μL from each serum sample to create PQC sample, vortex to mix well；

2. System Suitability Test (SST) sample preparation: Dilute the internal standard working solution five times with a 10% methanol/90% water solution (standard solution) as the SST sample;

3. Blank solution: Use extraction solvent as a blank solution (methanol pre-cooled in a -80°C freezer for more than half an hour)；

4. Take 20μL supernatant, 20μL PQC, and 20μL blank solution each in a 2ml centrifuge tube, add 20μL internal standard working solution, then add 110μL extraction solvent (methanol pre-cooled in a -80°C freezer for more than half an hour), vortex and shake for 1 minute; place at -20°C for at least half an hour.

5. Centrifuge at 14000xg, 4°C for 10 minutes, transfer the supernatant to a new 2ml centrifuge tube, vacuum freeze-dry, resuspend in 100μL of 10% methanol/90% water solution (standard solution), vortex for 30 seconds, sonicate for 1 minute, centrifuge at 14000xg, 4°C for 10 minutes, transfer the supernatant to a sample vial for mass spectrometry analysis;

Conditions for Chromatography

1.Simultaneous collection was performed in both positive ion mode and negative ion mode using a Waters ACQUITY BEH C18 column (1.7 µm × 2.1 mm × 100 mm).

2.The mobile phase consisted of A: 0.1% formic acid in water and B: 0.1% formic acid in acetonitrile/methanol (40/60) solution.

3.The column temperature was maintained at 40°C, with an injection volume of 5 μL, and gradient elution conditions as follows:

| Time (min) | Flow (ml/min) | A (%) | B (%) |
| --- | --- | --- | --- |
| 0 | 0.25 | 90 | 10 |
| 3 | 0.25 | 60 | 40 |
| 5 | 0.25 | 5 | 95 |
| 8 | 0.6 | 0 | 100 |
| 10 | 0.6 | 0 | 100 |
| 10.5 | 0.25 | 90 | 10 |
| 13.5 | 0.25 | 90 | 10 |

LC-MS Analysis

Metabolites were separated using a Waters ACQUITY BEH C18 Column (1.7 µmx2.1 mm x100 mm) on a Vanquish Flex UPLC equipped with a refrigerated autosampler (10°C) and column heater (40°C).

Two moblie phase condition was used to improve the metabolite coverage. For condition 1, solvent A (0.1% formic acid in water) and solvent B (0.1% formic acid in acetonitrile/methanol=4/6) were used to elute the metabolites with a 13.5 min gradient, as follows: 10% B at 0 min, 0.25ml/min; 40% B at 3 min, 0.25ml/min; 95 % B at 5 min, 0.25ml/min; 100 % B at 8 min, 0.6ml/min; 100 % B at 10 min, 0.6ml/min; and back to 10 % B at 10.5 min, 0.25ml/min; and equilibrate for 3min. Samples were analyzed using a Q Exactive HF-X (QE-HF-X) mass spectrometry equipped with a heated electro-spray ionization (HESI) source. All the data was acquired in positive and negtive switching mode using Full scan detection, and the PQC were also analyzed with Full scan/ddMS2 to acquire MS2 fragementation for metaoblite identificaiton and annotation. For conditon 2, solvent A was 6.5mM NH4HCO3 in water, solvent B was 6.5mM NH4HCO3 in methanol. The 12 min gradient was employed: 0 min, 0.25ml/min, 10% B; 4 min, 0.25ml/min, 40%B; 6 min, 0.25ml/min, 95%B; 8 min, 0.4ml/min, 99%B; 8.5 min, 0.4ml/min, 99%B; 8.6 min, 0.25ml/min, 10%B; 12 min, 0.25 ml/min, 10%B. Data acquisiton were performed only in negative mode, and Full scan was used for all the samples, and Full scan/ddMS2 was also used for PQC samples.

The Full Scan settings were as follows: 60,000 resolution, AGC target, 1e6; Maximum IT, 100 ms; scan range, 60 to 900 m/z. For Full scan/ddMS2(DDA), Top 20 MS/MS spectral (dd-MS2) @ 15000 were generated with AGC target = 2e5, Maximum IT=25 ms, and (N)CE/stepped NCE = 10, 40, 80v. Metabolites detection and identification were performed using MS-dial (ver.5.1.230912) by searching against online database (MoNA, GNPS, HMDB and MS-dial database) and in-house database.
